# What deters GPs from talking about sexual assault? a qualitative interview study

**DOI:** 10.3399/BJGP.2024.0761

**Published:** 2025-09-23

**Authors:** Fiona Elizabeth van Zyl-Bonk, Antoinette Leonarda Maria Lagro-Janssen, Anna Maria Cornelia Henrica de Klein, Theodora Alberta Maria Teunissen

**Affiliations:** 1 Department of Primary and Community Care, Research Institute for Medical Innovation, Radboud University Medical Center, Nijmegen, the Netherlands; 2 Research Institute for Medical Innovation, Radboud University Medical Center, Nijmegen, the Netherlands

**Keywords:** communication barriers, biomedical gaze, gender role, general practice, sexual assault, solutionism

## Abstract

**Background:**

Sexual assault affects overall health due to its somatic, psychological, and social consequences. Many survivors will present their health issues to GPs, unaware of the relationship between their symptoms and their history. Studies on intimate partner violence (IPV) show that GPs often lack the competencies to recognise and discuss IPV, which leads to substandard care. This leads to the research question of whether this also applies to sexual assault.

**Aim:**

To explore GPs’ experiences regarding the identification and discussion of sexual assault.

**Design & setting:**

Qualitative study using semi-structured interviews with GPs in the Netherlands.

**Method:**

Interviews were conducted with 14 GPs between March and August 2023. The interviews were transcribed and analysed using thematic analysis.

**Results:**

Most GPs thought sexual assault should be discussed in their consulting rooms. Although both barriers and facilitators were explored, GPs talked predominantly about barriers. Professionally, they felt hampered by the tendency to approach symptoms from a biomedical perspective and by their fixation on problem solving. Emotional resistance and discomfort also prevented them from discussing sexual assault. Female doctors struggled to remain aware that males can also be victims of sexual assault, and male doctors were reluctant to discuss sexual assault with female victims because of a fear of doing harm.

**Conclusion:**

This study provides insights into why GPs find it hard to talk about sexual assault. The findings indicate that they should be offered training that tackles the issues raised in order to become competent at recognising and discussing sexual assault with survivors.

## How this fits in

Previous research on sexual assault has predominantly focused on mental, sexual, and reproductive health care. As sexual assault affects overall health, the identification of survivors is especially needed in the context of primary care. Future training of GPs on talking about sexual assault is advisable and should incorporate reflections on their problem-solving outlook from a biomedical point of view, as well as on their potential personal emotional reluctance to put the topic on the agenda. Providing helpful resources, such as a social map/potential support network and opportunities for consulting experts in this field, is also important to facilitate discussions about sexual assault.

## Introduction

Sexual assault is a broad term that describes all forms of sexual behaviour in which someone engages an individual without consent. More than 53% of women and almost 19% of men have faced sexual assault during their lifetime;^1,2^ it is something that can impact overall health with physical, psychological, relational, and/or sexual sequelae that extend well beyond immediate harm.^3,4^ There is growing awareness that many patients seeking care from their GPs have a history of sexual assault.^5^

Several studies have reported associations between a history of sexual assault and chronic pain, as well as gastrointestinal, urogynaecological, and cardiopulmonary symptoms.^3,4,6–9^ Most studies were population based^3,4^ or were conducted in secondary care,^7,8^ but the association with urogynaecological symptoms was also found in studies in primary care.^5,6^ Sexual assault also leads to an increased risk of hospitalisation.^10^

Despite these impacts, sexual assault is often not a topic of conversation in the consultation room. Survivors do not disclose their history for reasons relating to shame, post-traumatic stress symptoms (such as avoidance of painful memories), and for fear of being blamed or not being believed.^5,11–13^ Additionally, they are often unaware of the relationship between their history and their current symptoms.^13^ GPs, for their part, are not always aware of the huge prevalence of sexual assault and its disastrous lifelong impact, or they may feel unable to act.^2^ Studies on discussing intimate partner violence (IPV) and childhood trauma in primary care revealed that health professionals are often disinclined to do so due to inadequate training and personal discomfort;^14–16^ as a result, as few as 8% of survivors disclose to professional care practitioners, including GPs;^17^ this figure rises to >50% when patients are explicitly asked about their negative sexual experiences.^18^

The lifelong physical consequences of sexual assault can be explained by the ongoing and dysfunctional interaction between cortisol and the immune system mediated by the hypothalamic-pituitary-adrenal axis, as a result of persisting stress due to trauma.^19^ Dysregulation of the immune system is a known cause of autoimmune and chronic diseases.^20^ Almost half of survivors of child sexual assault are sexually victimised again in the future^21^ so, when prior victimisation remains untreated, this could lead to even more morbidity over time.

More than any other specialty, primary care providers are in a unique position to identify signs and patterns associated with sexual assault. They are also well equipped to coordinate appropriate care as they act as gatekeepers to specialist care and maintain longstanding relationships with their patients. Based on studies concerning IPV we hypothesise that addressing sexual assault leads to more-effective consultations and improves quality of care and patient outcomes.^14,22,23^ In practice, however, healthcare providers often fail to raise the issue.

The aim of this study was to explore the experiences of GPs regarding the identification and discussion of sexual assault. It is hoped that providing additional knowledge on this topic will serve to guide the development of training programmes that will:

- better equip GPs to respond to, and manage, the health issues that accompany sexual assault, and
- help prevent revictimisation.

## Method

### Study design

A qualitative approach was chosen to explore GPs’ experiences of identifying and discussing sexual assault.

### Participants and recruitment

Seventy-three Dutch GPs from the research team’s national network called UgynHAG were contacted by email to help with the recruitment process, and a letter containing information about the study was provided to them. This network consists of GPs with a special interest and training in urogynaecology as approved and facilitated by the Dutch College of General Practitioners (NHG); as they have more knowledge of sexual assault than the average GP, they were asked not to participate themselves but to ask a GP from their local network who did not have special knowledge of sexual assault to do so. Twenty-six of them voluntarily accepted the request; this yielded the names of 21 GPs who expressed an interest in participating.

An online survey was sent to the 21 GPs so that background information could be collected, and a purposive sample was selected based on age, gender, cultural background, work experience, and region of practice. Initially, 10 GPs were contacted and interviews with them were arranged. An additional six GPs were then selected based on a variety of characteristics. Of the 16 GPs selected for the purposive sample, two declined to participate despite their earlier commitment: one stated a lack of time, the other did not give a reason. When data saturation was reached, the remaining five GPs were not included. An informed consent form was signed by all participants and confirmed orally preceding the interview.

### Data collection

Data were collected through semi-structured interviews by telephone or videoconference. The interviews were conducted by a trained female interviewer between March and August 2023. Based on the aim of the study, and with the help of the literature and research team expertise, an interview guide (Box 1) was created. After assessing a pilot interview — which was not included in the dataset — the research team made small adjustments to better suit the exploratory character of the study. The guide contained open-ended questions concerning the GPs’ experiences of identifying and discussing histories of sexual assault and their perceptions. Participants received no rewards or incentives.

Interview duration ranged between 30 and 60 minutes. Data collection ended when no new relevant information emerged from the interviews. All interviews were digitally audio-recorded and transcribed verbatim by the interviewer, with the help of a medical student. Transcripts were not returned to the participants.

Box 1. **Interview guide**

1. In the accompanying letter, we mentioned that sexual assault is often not talked about by the patient or by the GP. Is this something you recognise from your own experience? Why do you or do you not?
2. When are you reminded that a patient might possibly have experienced sexual trauma?
  - Do you have a concrete example?
  - What helps you to think of it?
  - What prevents you from thinking of it?
  - Do you sometimes think of it, only to reject the thought immediately? If so, would you care to explain? Do you have a concrete example?
3. You suspect that a patient (male or female) consulting with you may have experienced sexual assault. What do you do?
  - Do you have a concrete example?
  - When you decide to discuss sexual assault, what factors are helpful or have helped you? Why?
  - Have you experienced impediments in discussing this topic? If so, what were these impediments?
  - Have you ever wanted to do things differently in hindsight? If so, what? What might be the reason for this?
  - Are there any grounds for you not to discuss sexual assault? Do you have a concrete example?
4. What, in your view, is the prevailing attitude among GPs on this topic?
5. Is there anything you would like to add?

### Data analysis

The transcripts were analysed by two independent researchers, with the aid of the data analysis program ATLAS.ti (version 23). Drawing on the principles of grounded theory, data were analysed using thematic analysis.^22,24^ After familiarising themselves with the data, both researchers carried out preliminary coding of the first two interviews. They then independently organised the emerging themes into clusters, based on discussion and consensus with the entire research team. This initial framework was then applied to the remaining data. Codes were compared and discussed several times among the research team, and modified as more codes emerged. After implementation of the final framework on the complete dataset, and exploration of the relationships between concepts and themes, the themes were further refined in agreement with the research team.

The conception and conduct of this study were in accordance with the consolidated criteria for reporting qualitative research.^25^ Quotations are followed by the participant’s number and gender, and have been translated from Dutch into English by a professional translator.

## Results

Fourteen GPs participated, eight of whom were female (Table 1,1). No new codes or concepts emerged after 12 interviews. Participants varied in age, years of GP work experience, and cultural background. Approximately one-third of GPs had previously attended a lecture on sexual assault; the answers they gave did not differ from those who had not attended a lecture.

**Table 1.** Participants’ characteristics

Characteristic	*n*
**Gender**	
Male	6
Female	8
**Age, years**	
<40	4
40–55	5
>55	5
**Experience as GP, years**	
GP registrar	1
<5	1
5–15	6
>15	6
**Practice partner status**	
Partner	7
Locum GP	5
Employed	2
**Cultural background**	
Dutch	10
Other	4
**Region of practice location**	
North	4
East	5
South	2
West	3
**Area of practice location**	
City	7
Countryside	7
**Attended lecture on sexual assault**	
Yes	5
No	9

The most important facilitating factors mentioned were the awareness that sexual assault occurs in patients, practice-oriented training, and experience of discussing sexual assault that was gained over time:

*‘Well, awareness helps a lot ... ‘Cause if there’s no awareness, then it tends to pass you by quite easily and you’ll fail to pick up the signals, I think. And the signals, of course, are never specific ... So it’s non-specific signals you should be alerted to, and you need to be always willing and able to pick them up because of awareness ... This has helped to make me more alert.’* (Participant [P]4, male)

In addition, having access to services and support were seen as enablers for raising the issue:

*‘... I should engage in a little more training and role play so as to have more language at my disposal ... knowing the sexual assault centre, knowing what your helplines are — these things have helped me.’* (P3, female)

During analysis, however, it became clear that the interviewees, essentially, revealed impeding factors, which resulted in two main themes:

- professional pitfalls regarding a doctor’s biomedical gaze, solutionism, and dismissal or shifting of responsibility; and
- personal and interpersonal challenges regarding patient–physician gender differences and emotional resistance.

### Professional pitfalls

#### Biomedical gaze

GPs remarked on their tendency to mainly look at symptoms from a biomedical point of view, thereby overlooking other sources of origin:

*‘Only, yeah, sometimes you’re simply in a tunnel vision during a conversation and then you think, “It’s about those bile ducts” or “It’s about those intestinal problems” and then I am not thinking about … sexual assault as a factor.’* (P12, male)

Thinking about why discussing sexual assault is so difficult, interviewees realised that they were trained in medicine as biomedical doctors:

*‘I guess you’re terribly focused on putting somatic things first … And so what may be an impediment, perhaps, is that you’re simply over-focused on a somatic approach.’* (P14, female)

After they had first eliminated all potential physical causes, GPs tended to consider what else might be causing their patient’s ailment. Potential psychosocial causes, for example, easily took a back seat if symptoms could be attributed to a somatic cause. In particular, when confronted with many different physical symptoms at once, GPs found it challenging to look beyond the scope of somatic diagnoses:

*‘I think I mostly miss it when there are many somatic complaints that can, of course, be partly explained by this issue. Then that’s somewhat of a pitfall that you don’t think of it then, or you don’t ask about it, and the patients aren’t sending any signals either.’* (P1, male)

In addition, when an underlying medical condition had finally been ruled out, some GPs were more inclined to opt for a symptomatic treatment than to explore other possible explanations, thereby further increasing the likelihood of a history of sexual assault going unnoticed:

*‘Together, you embark on a path full of all these medically unexplained symptoms and you think, “Let’s do one more referral for the pain”, and* [then] *yet another one.’* (P8, female)

#### Solutionism

Interviewees said that their focus on trying to solve the issue prevented them from thinking of the possibility of sexual assault, especially when they were inexperienced:

*‘I was just like that at first, you know: “How can I help her?” I’ve got a full-on problem-solving mindset, and so my mind was wondering “how can I solve this?”’* (P9, male)

They supposed that a conversation about sexual assault would only be helpful and appropriate if they, as the GP, had a solution to offer in return. This could lead to a tendency to not address the topic of sexual assault at all:

*‘Look, if things can be more or less resolved and run a fairly normal course, then you will never raise the issue.’* (P6, female)

Interviewees indicated they did not really know what they could offer a victim of sexual assault after disclosure:

*‘Well, for me, it’s an impediment if I’m not sure what steps to take afterwards ... For I think I wouldn’t be sure now where I’m allowed to take this ... So this is not so much about getting the issue out in the open, but about conducting a proper conversation that’ll serve to help the patient.’* (P10, female)

Faced with an ever-increasing workload and time constraints, GPs said that adopting a solutionistic mindset appeared to be the best way of coping with the strain:

*‘Time. The rush during consultations, running late. Which makes you think, “Let’s just focus on the complaint someone presents* [with] *and let’s not think too far outside the box”. Yes, maybe* [there is] *too much focus on the complaint itself.’* (P11, male)

#### Dismissal or shifting of responsibility

Awareness of sexual assault was considered too much to expect from GPs on top of their increasing workload. According to these participants, sexual assault was a social problem rather than a health concern:

*‘It’s not our job, but it’s social work … The attitude* [among GPs] *is … that it’s not our core business.’* (P9, male)

It was thought that, if GPs stuck to their responsibility of referring patients, these questions would automatically emerge during any mental health assessment. One participant stated that:

*‘… such people* [those with medically unexplained symptoms] *are referred for a multidisciplinary approach, and there’s also often a psychologist on the team.’* (P10, female)

### Personal and interpersonal challenges

#### Patient–physician gender difference

Female GPs acknowledged that they found it harder to identify a history of sexual assault in a male patient; they cited unawareness of male victimisation, lack of training on specific signs in males, and fear of embarrassing the patient as reasons for this:

*‘I think I’m much more alert with women than with men … for some reason, my alarm bells don’t go off as quickly … just because I have had less experience with it.’* (P4, female)

According to male doctors, their female patients, in particular those who experienced sexual assault, would rather see a female GP; this would lead to male GPs having less exposure to, and experience with, survivors of sexual assault. Male GPs felt more hesitant to discuss sexual assault with female patients compared to male patients. In addition, they felt more hesitant to discuss it with their female patients than they assumed their female counterparts would. Being males in an authoritative position, they feared that if they brought up the topic, they would remind the patient of their (presumably male) perpetrator and trigger a harmful response. It was not mentioned whether this view also extended to male survivors of sexual assault:

*‘*… *I find it hard to have their full trust … I think it’s easier when two women talk about such sexual experiences than when there is a man sitting across from you … Yes, my feeling is that they don’t easily, well, reveal everything because, at the end of the day, I’m still a man.’* (P9, male)

#### Emotional reluctance

Although GPs acknowledged the importance of discussing sexual assault, many found it hard to put this into practice because of the negative emotional response evoked by the topic:

*‘… sexual violence is a painful and uncomfortable subject for people in any case — both for patients and for GPs.’* (P6, female)

GPs admitted that they personally experienced this apprehension, which, in one case, resulted in a GP not addressing the issue of sexual assault, even though there was a suspicion of it having taken place:

*‘… especially at first, I didn’t dare to ask or I lost myself in endlessly referring people, while I actually thought this was going on.’* (P8, female)

During the interviews, some became more consciously aware of their emotional recoil from the subject, which had made them oblivious to sexual assault as a possible issue for their patients:

*‘I think I have a blind spot for it … Yeah, I don’t know where that comes from. I guess that you just don’t want to think about it.’* (P2, male)

What often accompanied this feeling was the fear of directly doing harm to victims or to their recovery process. This fear increased when an attempt to talk about sexual assault was met with resistance from the patient. GPs felt more inhibited when they noticed their patients’ reluctance to talk about it, which often seemed to be the case:

*‘Well, I notice often that people get defensive … Then I would rather not proceed, like, “Oh dear, I’m going somewhere where things are not, not comfortable”.’* (P6, female)

In a few cases, the GPs’ personal background added to their inclination to avoid or dismiss the subject; this could be because they felt they were hitting too close to home or were too far from their frame of reference:

*‘In my own family there was also incest, you know, so there are all these unpleasant thoughts attached. So, I guess that also inhibits me* [from talking about it]*.’* (P13, female)

*‘I come from an environment where this never happens, you know. And I think that most doctors have not* [personally] *experienced this either.’* (P9, male)

## Discussion

### Summary

GPs reflected on challenges and struggles in identifying and addressing sexual assault. They tended to fit their patients’ medical problems into a biomedical frame, thereby easily overlooking important potential psychosocial causes, such as a history of sexual assault. Raising the subject became even less likely when, partly encouraged by workload pressures, or a dismissal, or shifting of responsibilities, GPs adopted a solutionistic attitude.

Sexual assault also challenged GPs on a personal level, evoking discomfort in general, or hesitancy or fear of doing harm when they faced a survivor of the opposite sex and when up against their own emotional resistance. The key factors that GPs felt facilitated their raising the issue of sexual assault were: being aware that it occurs in patients, having received training about sexual assault, having experience of discussing sexual assault that had been gained over time, and having access to services and support.

### Strengths and limitations

To the authors’ knowledge, this study is the first to explore GPs’ experiences regarding awareness of, and communication about, sexual assault in primary care. The sample included GPs who varied in age, gender, work experience, and cultural background, and who worked in various geographic settings throughout the Netherlands. The interviews were conducted by an experienced interviewer, and the research team consisted of practising GPs with ample expertise on research methods and the subject of sexual assault; this led to rigorous analysis of the data and ensured the credibility of the identified themes.

The study did, however, also have some limitations. Utilising the authors’ own network of GPs to help with the recruitment process was a possible biasing factor. In addition, as the subject of sexual assault was known by potential participants beforehand, and is an issue that may cause discomfort, it is possible that those GPs who were selected had — at least partly — overcome any such feelings. This raises the question of whether the study findings can be extrapolated to the general population of Dutch GPs. Although efforts were made to minimise potential effects, the answers GPs presented might have been subject to social desirability bias. In reality, therefore, the ‘blind spots’ to disclosing sexual assault and the obstacles to discussing sexual assault could be much greater.

### Comparison with existing literature

Previous research on addressing sexual assault has mainly targeted mental and sexual health care, and especially from the viewpoint of the survivor, not the care provider. The sparse studies on perceived barriers among physicians, however, also identified discomfort as a major barrier to inquiry.^15,26^ The conclusion of a disturbingly large and serious Dutch sexual assault case is that the most impeding factor for identification and action of anyone involved is the natural tendency to look away from the possibility of it having happened and dismiss its signals.^27^ Disengaging from a distressing subject such as sexual assault is an instinctual response to protect oneself from further emotional distress.^28^

The results presented here resonate with an extensive body of knowledge on IPV, including competing priorities,^29,30^ lack of awareness,^29,30^ deferring responsibility,^31^ fear of doing harm,^31^ personal discomfort,^29,32,33^ and the caregiver’s male gender.^14^ In this discussion, the focus is on four themes: the biomedical gaze, a solution-oriented approach, the gender dynamics between patient and GP, and emotional reluctance.

Studies on IPV have confirmed that trying to fit physical complaints into a biomedical gaze alone will fail to detect important psychosocial causes and will ignore the emotional needs of patients.^30,31^ This is closely tied to somatic fixation, a process whereby physicians and/or patients focus exclusively — and inappropriately — on the somatic aspects of a complex problem.^34^ This may occur with any illness, but is seen particularly with somatoform disorders and psychosomatic diseases. As this study shows, it is important for GPs, therefore, to go beyond biopathology and to integrate a biopsychosocial approach. Studies about medically unexplained symptoms have also shown doctors’ difficulties in understanding the mind and body as integrated, rather than separate, entities.^7,35^ Seeing the mind and body as integrated with each other will help to address the need to find organic causes for patients’ symptoms and encourage a holistic approach to care.^36^

A solution-oriented approach, as the findings of this study affirm, could further prevent GPs from exploring a possible experience of sexual assault. As it involves perceiving the world in terms of problems that are in need of a technological solution,^37^ presuming that they can be solved, solutionism can be both a cause and an effect of somatic focus.^34^ What emerged from studies on IPV is that physicians’ perceptions of their role as problem solvers made them disinclined to assess for IPV as there was no ‘quick fix’ for that in an environment dominated by time pressure and priorities.^29–32^ This could equally be true for sexual assault. Early enquiry into a history of sexual assault, however, can facilitate a correct diagnosis and appropriate treatment earlier on in the process, ultimately saving time and resources.^7^

The authors of the study presented here will also underline the existing gender dynamics in patient–doctor communication. Research indicates that female patients often feel more comfortable disclosing sexual abuse to female providers.^38^ Male patients may face barriers due to societal norms on masculinity that may complicate disclosure.^14,39–42^ Female doctors tend to engage in more emotionally charged interactions,^40^ which may facilitate the disclosure process. In contrast, male doctors may inadvertently create barriers to disclosure, due to their more distant approach and societal norms that discourage emotional expression in male patients;^40^ this, together with reducted awareness that sexual assault also occurs in males, means that sexual assault in males is much less recognised and discussed.^41^ Ultimately, the effectiveness of talking about sexual assault is predominantly affected by the provider’s ability to create a supportive and understanding environment, regardless of the patient’s gender.^43–46^

Lastly, this study shows that a physician’s personal history can affect the comfort level of talking about sexual assault. A study on personal exposure to sexual assault among midwives revealed a prevalence that does not differ from the general population.^47^ Then we can assume this applies to GPs as well. In this group, especially, discussing sexual assault could be difficult; however, research on this topic concluded that ‘shared trauma’ can also facilitate identification.^47^ Nevertheless, GPs without a history of sexual assault may also find it to be an emotionally demanding subject, as is known from a study on IPV.^44^

### Implications for practice

In the case of IPV, a 1.5-day training programme for GPs has been proven to increase the identification of partner abuse in women by up to 4.5 times.^48^ It improves awareness of abuse, leads to active questioning of women about IPV, and results in increased identification of abuse in women. It is likely that such a training programme could also be used to train GPs in how to diagnose and discuss a history of sexual assault. Such a programme should increase awareness and pay attention to a biopsychosocial approach, as well as to the under-recognition of male victims and the emotional impact of sexual assault on the GPs themselves. It should also provide tools on how to ask about sexual assault, and offer GPs and patients options to obtain services and support; some tips for practice are outlined in Box 2. Aside from training, it is important to make sure that GPs are aware of the social map and opportunities that exist for consulting experts in this field.

Box 2. **Practice tips**

- Be aware that >50% of women and almost 20% of men have experienced sexual assault during their lifetime.
- When asking about sexual assault, create a safe and supportive environment.
- Ask about sexual assault in a sensitive way.
- Use open-ended, non-threatening questions, such as:
*‘I know from experience that patients with complaints like the ones you mention have often gone through a traumatic event, such as physical or sexual abuse. Might this be a factor in your case too?’*
*‘Because we know that many women/men go through physical and sexual abuse and this may cause a variety of physical and mental complaints, we now routinely ask about sexual abuse. Have you ever had to deal with that?’*
- Consider sexual assault as a possible underlying factor in chronic unexplained symptoms and be proactive about asking questions.
- Know your local support network (social map).
- Follow up and ensure continued support is provided.

A review of training methods showed that recognition and discussion of past sexual assault by healthcare providers can be effective if an alternating mix of multiple active and passive training methods are used, with room for feedback and personal experiences.^48^ Appropriately trained GPs will help to alleviate the health consequences of sexual violence and reduce its recurrence.^16^

Talking about sexual violence is challenging for all parties involved. In addition, professional perspectives and personal challenges can hamper GPs’ awareness and determination to raise the subject during consultations. If, however, GPs learn to expand their view beyond a biomedical and solutionistic outlook, and can acknowledge their personal or gender-related reasons for hesitancy and emotional reluctance, this will enable them to offer victims of sexual assault better and more complete care.

## Data

Dataset is not publicly available.
